# Changes and related factors of blood CCN1 levels in diabetic patients

**DOI:** 10.3389/fendo.2023.1131993

**Published:** 2023-06-02

**Authors:** Zhao-Yu Xiang, Shu-Li Chen, Xin-Ran Qin, Sen-Lin Lin, Yi Xu, Li-Na Lu, Hai-Dong Zou

**Affiliations:** ^1^ National Clinical Research Center for Eye Diseases, Department of Ophthalmology, Shanghai General Hospital, School of Medicine, Shanghai Jiao Tong University, Shanghai, China; ^2^ Shanghai Engineering Center for Precise Diagnosis and Treatment of Eye Diseases, Shanghai Eye Diseases Prevention & Treatment Center, Shanghai Eye Hospital, Shanghai, China

**Keywords:** diabetic Retinopathy, CCN1/Cyr61, diabetic complications, blood-retinal barrier, blood biomarker

## Abstract

**Objective:**

To study the differences in blood cellular communication network factor 1 (CCN1) levels between patients with diabetes mellitus (DM) and healthy individuals and to explore the relationship between CCN1 and diabetic retinopathy (DR).

**Methods:**

Plasma CCN1 levels were detected using ELISA in 50 healthy controls, 74 patients with diabetes without diabetic retinopathy (DM group), and 69 patients with diabetic retinopathy (DR group). Correlations between CCN1 levels and age, body mass index, mean arterial pressure, hemoglobin A1c, and other factors were analyzed. The relationship between CCN1 expression and DR was explored using logistic regression after adjusting for confounding factors. Blood mRNA sequencing analysis was performed for all subjects, and the molecular changes that may be related to CCN1 were explored. The retinal vasculature of streptozotocin-induced diabetic rats was examined using fundus fluorescein angiography; in addition, retinal protein expression was examined using western blotting.

**Results:**

Plasma CCN1 levels in patients with DR were significantly higher than in the control and DM groups; however, no significant differences were observed between healthy controls and patients with DM. CCN1 levels negatively correlated with body mass index and positively correlated with the duration of diabetes and urea levels. It was observed that high (OR 4.72, 95% CI: 1.10–20.25) and very high (OR 8.54, 95% CI: 2.00–36.51) levels of CCN1 were risk factors for DR. Blood mRNA sequencing analysis revealed that CCN1-related pathways were significantly altered in the DR group. The expression of hypoxia-, oxidative stress-, and dephosphorylation-related proteins were elevated, while that of tight junction proteins were reduced in the retinas of diabetic rats.

**Conclusion:**

Blood CCN1 levels are significantly elevated in patients with DR. High and very high levels of plasma CCN1 are risk factors for DR. Blood CCN1 level may be a potential biomarker for diagnosis of DR. The effects of CCN1 on DR may be related to hypoxia, oxidative stress, and dephosphorylation.

## 1 Introduction

Diabetes is a metabolic disease characterized by hyperglycemia. Long-standing hyperglycemia chronically damages various organs, particularly the eyes, kidneys, heart, blood vessels, and nerves, leading to serious complications. Diabetic retinopathy (DR) is one of the most common microvascular complications of diabetes and one of the leading causes of incident blindness in working-age adults (1).

Cellular communication network factor 1 (CCN1) is a dynamically expressed extracellular matrix protein (2). CCN1, which is primarily secreted by endothelial cells, binds to the extracellular matrix and cell surface and controls the signal transduction between them (3). CCN1 is rapidly upregulated in response to various stimuli (e.g., mechanical/shear stress, hypoxia, and inflammation) and plays a key role in wound repair, cell growth, differentiation, adhesion, and angiogenesis (4). CCN1 is an essential regulator of vascular development and an important component of pathological neovascularization.

CCN1 is closely associated with diabetic microangiopathy (5–8), particularly diabetic retinopathy (5). You et al. reported the occurrence of higher levels of CCN1 in the vitreous humor of patients with proliferative diabetic retinopathy (PDR) compared to patients without diabetes (5). This observation has been consistently confirmed by Zhang et al. (9), Choi et al. (10), and Zhou et al. (11). Additionally, it was observed that the vascular activity in patients with PDR significantly affected the vitreous levels of CCN1 (11, 12). Zhou et al. observed that CCN1 levels were significantly higher in patients with active PDR than in patients with quiescent PDR (11); additionally, Zhang et al. found that vitreous CCN1 levels were higher in patients with PDR who had extensive neovascularization in the retina than in those with limited neovascularization (9). However, CCN1 levels were higher in the PDR group than in the control group, regardless of the PDR status (11, 12). Furthermore, CCN1 was upregulated in the neovascularized membranes of patients with PDR (12, 13) and was mostly expressed in endothelial cells and myofibroblasts (12). Additionally, the anti-vascular endothelial growth factor (VEGF) vitreous injection was able to reduce VEGF levels in the vitreous; however, it was unable to reduce CCN1 levels (12).

Animal and in vitro experiments have demonstrated the key role of CCN1 in DR (3–5, 9, 10, 13, 14). In animal models with streptozotocin (STZ)-induced diabetes, retinal CCN1 protein levels were significantly elevated (4, 9, 13, 15); additionally, Zhang et al. found that CCN1 was substantially concentrated in the ganglion cell and inner plexiform layers (9). Vitreous injections of exogenous advanced glycation end products and VEGF increased retinal CCN1 levels (4, 14). Depletion or knockdown of CCN1 significantly inhibited retinal neovascularization in mouse models with oxygen-induced retinopathy (3, 5, 10, 16, 17). Lee et al. reported that CCN1 produced by pericytes was capable of promoting choroidal neovascularization through Wnt5a (3). Di et al. found that the neoangiogenic effect of CCN1 was mediated by the PI3K/Akt-VEGF pathway (16, 17).

DR is a common microvascular complication of diabetes that can lead to vision loss and blindness; thus, the early detection and treatment of DR are crucial. The study of DR biomarkers is conducive to the early detection of DR and the exploration of its mechanism, as blood samples are easier to obtain than vitreous humor samples. Although CCN1 is closely associated with DR, there are few studies on blood CCN1 levels in patients with diabetes. Studies on the comparison of the blood CCN1 levels in patients with diabetes and healthy individuals and the relation between blood CCN1 levels and DR have not been reported. Additionally, the study of blood CCN1 levels, as a DR biomarker, could be a potential diagnostic tool. In this study, we analyzed plasma CCN1 levels and mRNA sequencing results in 50 healthy controls and 143 patients with type 2 diabetes (74 without DR and 69 with DR) to explore the predictive potential of plasma CCN1 in the diabetic population and the plausible mechanism through which systemic CCN1 affects DR.

## 2 Materials and methods

### 2.1 Subjects and examination

The participants included in this study were recruited from the Shanghai Cohort Study of Diabetic Eye Disease (SCODE; clinicaltrials.gov identifier: NCT03665090). This study adhered to the ethical principles of the Declaration of Helsinki and was approved by the Ethics Committee of Shanghai First People’s Hospital (2013KY023). In 2003, SCODE established a health record for diabetic residents and began conducting annual assessments (18). In 2008, SCODE built a community remote DR screening system that combined digital fundus photography with Internet technology, which improved the efficiency of DR diagnosis (19). This study selected cross-sectional data from the SCODE study conducted between 2017–2018.

The examination methods for the SCODE study have been previously described (20). The inclusion criteria for this study were as follows: (1) diabetes mellitus (DM) diagnosed in the endocrinology department and (2) intraocular pressure in both eyes in the range of 10–21 mmHg. The exclusion criteria were as follows: (1) history of eye diseases other than DR, such as glaucoma, optic neuropathy, and hereditary eye diseases, among others; (2) history of eye surgery; (3) severe systemic diseases; (4) inability to cooperate with the examination; and (5) fundus images of poor quality. The diagnoses of type 1 and type 2 diabetes in this study were based on the diagnostic criteria for diabetes proposed by the WHO in 1999 (21). The diagnosis of DR was based on the International Clinical Classification of DR proposed at the 2002 International Ophthalmology Conference (22).

The researchers recorded basic information such as the date of birth, sex, height, weight, diabetes type, diagnosis time, and medical history of the research subjects. Venous blood samples were drawn from each subject, and tested for the levels of blood glucose, hemoglobin A1c (HbA1c), total blood cholesterol, and blood urea, among others. The best-corrected visual acuity was measured using an international standard visual acuity chart. The eyelids, conjunctiva, cornea, anterior chamber, iris, pupil, and lens were examined using slit-lamp biomicroscopy (SL130, Zeiss, Germany). Body Mass Index and Mean Arterial Pressure were calculated using the following equations:


$$Body\;Mass\;Index\;\left(BMI,\;kg/m^{2}\right)=weight\;\left(kg\right)÷height^{2}\left(m^{2}\right)$$

$$\begin{array}{c} Mean\;Arterial\;Pressure\;\left(MAP,\;mmHg\right)\\ =systolic\;blood\;pressure\;\left(mmHg\right)\times \frac{1}{3}\\ +diastolic\;blood\;pressure\;\left(mmHg\right)\times \frac{2}{3} \end{array}$$

### 2.2 Plasma CCN1

CCN1 concentrations in human plasma samples were determined using a commercially available ELISA kit (DCYR10, R&D Systems, USA) according to the manufacturer’s instructions (mean minimum detectable dose: 1.54 pg/mL, intra-assay precision: 2.3%, inter-assay precision: 6.4%).

### 2.3 mRNA sequencing

Total white blood cell RNA was isolated using the RNeasy Mini Kit (Qiagen, Germany). Paired-end libraries were synthesized using the TruSeq™ RNA Sample Preparation Kit (Illumina, USA) following the TruSeq™ RNA Sample Preparation Guide and sequenced on an Illumina NovaSeq 6000 (Illumina, USA). Library construction and sequencing were performed by Sinotech Genomics Co. Ltd. (Shanghai, China).

Paired-end sequence files (FASTQ) were mapped to the reference genome (GRCh38.100) using Hisat2 (Hierarchical Indexing for Spliced Alignment of Transcripts, version 2.0.5). The output SAM (sequencing alignment/map) files were converted to BAM (binary alignment/map) files and sorted using SAMtools (version 1.3.1).

Gene abundance was expressed as fragments per kilobase of exons per million reads mapped (FPKM). StringTie software was used to count the fragments within each gene and the TMM algorithm was used for normalization.

Differential mRNA expression was analyzed using the edgeR package. Differentially expressed RNAs with |log1.5(FC)| values > 1 and q-value< 0.05, were considered significantly different and retained for further analysis. In this study, we specifically analyzed genes that differed in both DR vs. DM and DR vs. control comparisons that changed in the same direction.

### 2.4 Animals

Animals were handled in accordance with the Association for Research in Vision and Ophthalmology Statement for the Use of Animals in Ophthalmic and Vision Research. A single intraperitoneal injection (40 mg/kg) of STZ was used to induce type 1 diabetes in adult Brown Norway rats weighing >200 g. Rats were fasted for 12 h prior to intraperitoneal injection. Rats of similar age and weight (n = 6) were used as the control group and were injected with equal amounts of sodium citrate buffer (0.1 mol/L). Body weight and blood glucose levels were measured before the injection and at 3, 4, and 8 weeks after the injection. Rats were considered diabetic if their blood glucose levels were >300 mg/dL three days after STZ injection (n = 6). Nine weeks after the injection, the control and STZ rats were sacrificed, and retinal tissue samples were collected from each eye (**Supplementary Figure 1**). Tissue samples were placed in liquid nitrogen and subsequently stored at −80°C for further experiments.

### 2.5 Fundus fluorescein angiography

The rats in each group were subjected to fluorescein fundus angiography (FFA) using Micron IV (Phoenix Research Labs, USA) 8 weeks after the induction of DM. The rats were intraperitoneally injected with sodium pentobarbital (40 mg/kg) for anesthesia, pupil dilation was performed with one drop of tropicamide (Santen Pharmaceutical Corporation, Japan), and the corneal surface was coated with methyl cellulose to keep it moist. During the FFA examination, the rats were intraperitoneally injected with 10% sodium fluorescein (0.001 mL/g, Fluorescite, Alcon, USA) for a quick examination.

### 2.6 Western blot analysis

Proteins were extracted from tissue homogenates. For Western blot analysis, protein samples were fractionated in 4–20% SurePAGE™ Precast Gels (M00657, GenScript Biotech Corporation, China) and transferred to nitrocellulose membranes. The analysis was performed with anti-CCN1 (ab228592, Abcam, UK), anti-CDC42 (2466T, Cell Signaling Technology, USA), anti-Claudin5 (4C3C2, Invitrogen, USA), anti-COX6c (ab150422, Abcam, UK), anti-CREB1 (R23983, ZEN- BIOSCIENCE, China), anti-HIF1α (ab179483, Abcam, UK), anti-NDUFα1 (15561-1-AP, Proteintech Group, USA), anti- SEPN1 (55333-1-AP, Proteintech Group, USA), anti-SHP1 (ab32559, Abcam, UK, anti-VEGFa (NB100-664, Novus Biologicals, USA), and anti-β-tubulin (2146S, Cell Signaling Technology, USA) antibodies. Immunoassays were performed using enhanced chemiluminescence (17046; ZEN-BIOSCIENCE, China), in accordance with the manufacturer’s instructions. Protein levels were determined by densitometric scanning of protein bands. Six retinas were used in each group, and the results for each retina were averaged from three replicates.

### 2.7 Statistical analysis

The Kolmogorov–Smirnov U test was used to test the normality of all variables. Normally distributed descriptive data were represented as the mean ± standard deviation, and non-normally distributed descriptive data were represented as the median (quartile 1, quartile 3). One-way analysis of variance was used to compare the between-group differences in normally distributed data. The Kruskal-Wallis test was used to compare the between-group differences for non-normally distributed data. The Mann-Whitney U test was used to compare differences in non-normally distributed data between the two groups. Spearman’s correlation test was used to analyze the correlation between parameters when the data were normally distributed. A binary logistic regression model was established with DR as the dependent variable (DR = 1, without DR = 0) to evaluate the relationship between the variables and CCN1. CCN1 was stratified according to its quartiles (low CCN1< 127.72 pg/mL, 127.72 pg/mL ≤ moderate CCN1< 173.85 pg/mL, 173.85 pg/mL ≤ high CCN1< 250.32 pg/mL, very high CCN1 ≥ 250.32 pg/mL). Age, sex (male = 1, female = 0), BMI, duration of diabetes, urea, MAP, fasting glucose, HbA1c, low-density lipoprotein, and total cholesterol levels were included in the multivariate model for confounding factor correction. The Bonferroni correction was performed for multiple uncorrected comparisons. Differences were considered statistically significant at P< 0.05. Statistical analysis was performed using SPSS 22 software (International Business Machines Corporation, America).

## 3 Results

### 3.1 Characteristics of the participants

A total of 193 participants (95 female, 98 male) were included in the analysis. No significant differences (all p > 0.05) in age, BMI, MAP, low-density lipoprotein, and total cholesterol levels were observed between the groups (control, DM, and DR). However, levels of fasting glucose (H = 66.62, p< 0.001), HbA1c (H = 64.47, p< 0.001), high-density lipoprotein (F = 3.28, p = 0.04), and urea (F = 9.73, p = 0.008) were significantly different in the three groups. The levels of fasting glucose (H = 24.39, p = 0.027) and HbA1c (H = 31.48, p = 0.001) of the DR group were significantly higher than those of the DM group, whereas the levels of fasting glucose (H = 59.21, p< 0.001) and HbA1c (H = 52.34, p< 0.001) of the DM group were significantly higher than those of the control group. Levels of high-density lipoprotein were significantly higher in the control group than in the DM group (p = 0.036). Urea levels were significantly higher in the DR group than in the control group (H = 32.33, p = 0.005). Furthermore, the duration of diabetes was significantly longer in the DR group than in the DM group (H = 13.28, p< 0.001; **Table 1**).

**Table 1:** Characteristics of the participants.

	CCN1, pg/mL^†^	Age, y^†^	Duration of diabetes, y^†^	Fasting glucose, mmol/L^†^	HbA1c, %^†^	Urea, mmol/L^†^	BMI, kg/m^2‡^	MAP, mmHg^‡^	High-density lipoprotein, mmol/L^‡^	Low-density lipoprotein, mmol/L^‡^	Total cholesterol, mmol/L^‡^
Control	152.7 (104.9, 193.7)	69 (67, 74)	N/A	5.35 (5.15, 5.94)	5.80 (5.70, 6.15)	5.41 (4.31, 6.26)	24.74 ± 3.62	96 ± 9	1.42 ± 0.39	3.05 ± 0.89	5.25 ± 1.02
DM	164.0 (99.3, 218.0)	69 (66, 73)	11.0 (4.0, 21.5)	6.93 (6.05, 8.31)	6.80 (6.25, 7.40)	5.74 (4.93, 6.84)	24.71 ± 3.56	95 ± 7	1.25 ± 0.33	2.90 ± 1.10	4.95 ± 1.26
DR	235.4 (157.4, 291.6)	71 (66, 75)	18.0 (11.6, 22.4)	8.19 (7.11, 9.60)	7.70 (6.90, 8.65)	6.10 (4.98, 8.00)	24.43 ± 2.47	97 ± 8	1.34 ± 0.35	2.90 ± 0.96	4.88 ± 1.05
H/F	23.67	2.35	13.28	66.62	64.47	9.73	0.18	0.97	3.28	0.4	1.73
p-value	<0.001^*^	0.31	<0.001^*^	<0.001^*^	<0.001^*^	0.008^*^	0.83	0.38	0.04^*^	0.67	0.18

CCN1, Cellular Communication Network Factor 1; BMI, Body Mass Index; MAP, Mean Arterial Pressure; HbA1c, Hemoglobin A1c; DM, Diabetes mellitus; DR, Diabetic Retinopathy; N/A, not applicable.

^†^skewed data analyzed using Kruskal-Wallis test; ^‡^ normal distributed data analyzed using one-way analysis of variance; ^*^ significant difference among groups, p< 0.05.

### 3.2 Plasma CCN1

Blood CCN1 levels were significantly different in the three groups (H = 23.67, p< 0.001). CCN1 levels in the DR group were significantly higher than those in the DM (H = 38.44, p< 0.001) and control (H = 43.75, p< 0.001) groups. However, no significant difference in CCN1 expression was observed between the control and DM groups (H = 5.32, p > 0.05; **Table 1**). In addition, similar levels of CCN1 were observed in both male and female subgroups (**Supplementary Materials**).

### 3.3 Influencing factors of CCN1

There were no sex differences in the CCN1 levels in the control (U = 304, p = 0.630), DM (U = 768, p = 0.201), or DR groups (U = 605, p = 0.895). CCN1 levels negatively correlated with BMI (r = –0.359, p< 0.001) and positively correlated with the duration of diabetes (r = 0.370, p< 0.001) and urea levels (r = 0.206, p = 0.041). CCN1 levels were not significantly correlated (all p > 0.05) with age (r = 0.103), MAP (r = −0.112), fasting glucose (r = 0.189), HbA1c (r = 0.190), high-density lipoprotein (r = 0.132), low-density lipoprotein (r = 0.141), or total cholesterol (r = 0.091) (**Supplementary Figure 2**).

### 3.4 DR and CCN1

**Figure 1:**
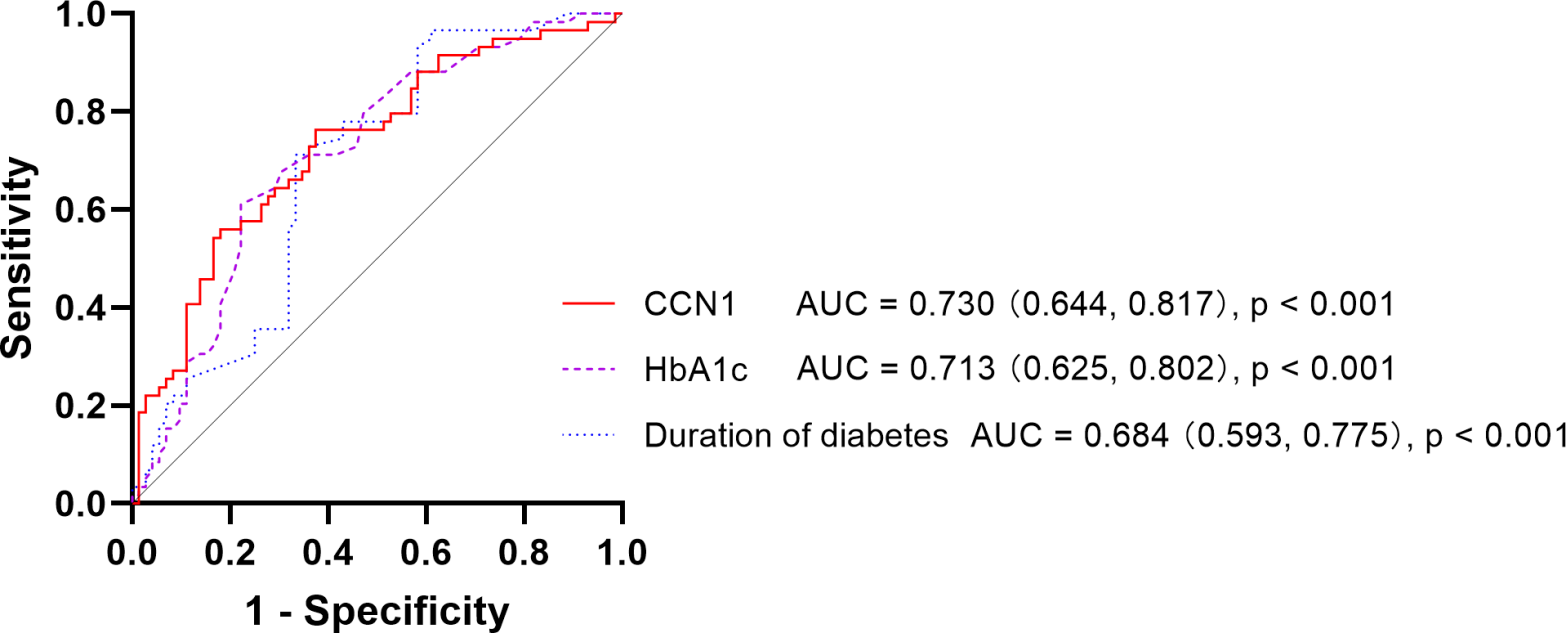
Receiver operating characteristic curve of DR DR, Diabetic Retinopathy; CCN1, Cellular Communication Network Factor 1; HbA1c, Hemoglobin A1c; AUC, Area Under Curve.

The areas under the receiver operating characteristic curves for DR were 0.730 (95% CI: 0.644–0.817), 0.713 (95% CI: 0.625–0.802), and 0.684 (95% CI: 0.593–0.775) for CCN1 levels, HbA1c levels, and duration of diabetes, respectively, whereas no significant differences were observed for age, urea, and BMI (all p > 0.05; **Table 2**; **Figure 1**).

**Table 2:** Association between CCN1 and DR: Binary and multivariable logistic regression analysis.

	Unadjusted model	Adjusted model 1^†^	Adjusted model 2^‡^	Adjusted model 3^§^
CCN1 low	Reference	Reference	Reference	Reference
CCN1 moderate	1.47 (0.51, 4.21)	1.49 (0.51, 4.30)	1.98 (0.53, 7.41)	2.28 (0.56, 9.34)
CCN1 high^*^	2.32 (0.86, 6.30)	2.41 (0.87, 6.63)	4.22 (1.10, 16.11) ^*^	4.72 (1.10, 20.25) ^*^
CCN1 very high^*^	5.91 (2.12, 16.49) ^*^	5.96 (2.11, 16.79) ^*^	8.99 (2.33, 34.62) ^*^	8.54 (2.00, 36.51) ^*^

CCN1, Cellular Communication Network Factor 1; OR, Odds Ratio; DR, Diabetic Retinopathy.

CCN1 was stratified according to its quartiles, low CCN1< 127.72 pg/mL, 127.72 pg/mL ≤ moderate CCN1< 173.85 pg/mL, 173.85 pg/mL ≤ high CCN1< 250.32 pg/mL, very high CCN1 ≥ 250.32 pg/mL

^†^adjusted for age and sex (male = 1, female = 0); ^‡^ adjusted for age, sex, BMI, duration of diabetes, and urea; ^§^adjusted for age, sex, BMI, duration of diabetes, urea, MAP, fasting glucose, HbA1c, high-density lipoprotein, low-density lipoprotein, and total cholesterol; ^*^significance of the logistic regression model, p< 0.05.

In patients with diabetes, very high blood CCN1 levels were associated with an increased risk of DR (OR 5.91, 95% CI: 2.12–16.49). Furthermore, after multifactorial correction, both high and very high blood CCN1 levels were found to be associated with an increased risk of DR (high: OR 4.72, 95% CI: 1.10–20.25; very high: OR 8.54, 95% CI: 2.00–36.51).

### 3.5 Changes in blood mRNA Associated with CCN1

Pairwise comparisons (DR vs. DM, DR vs. control) of genes obtained by mRNA sequencing analysis yielded 237 differentially expressed genes, of which 219 genes were upregulated and 18 genes were downregulated in the DR group. All the genes obtained from the screening met the |FC| value >1.5 and q-value<0.05. Among the 237 differential genes obtained by sequencing, those associated with CCN1 included *HIF1A-AS3, SELENON, MIF, CDC42-IT1, SRCAP, AC009927.1, AC116348.2, CDC42EP1, COX6CP1, AC112191.1, AL117336.1, NOG*, and *AL157871.3.*

Moreover, *HIF1A-AS3, SELENON, MIF, CDC42-IT1, SRCAP, AC009927.1, AC116348.2, CDC42EP1, COX6CP1, AC112191.1*, and *AL157871.3* were upregulated, while *AL117336.1* and *NOG* were downregulated in the DR group (**Supplementary Tables 1, 2**).

### 3.6 Animal examination of FFA

**Figure 2:**
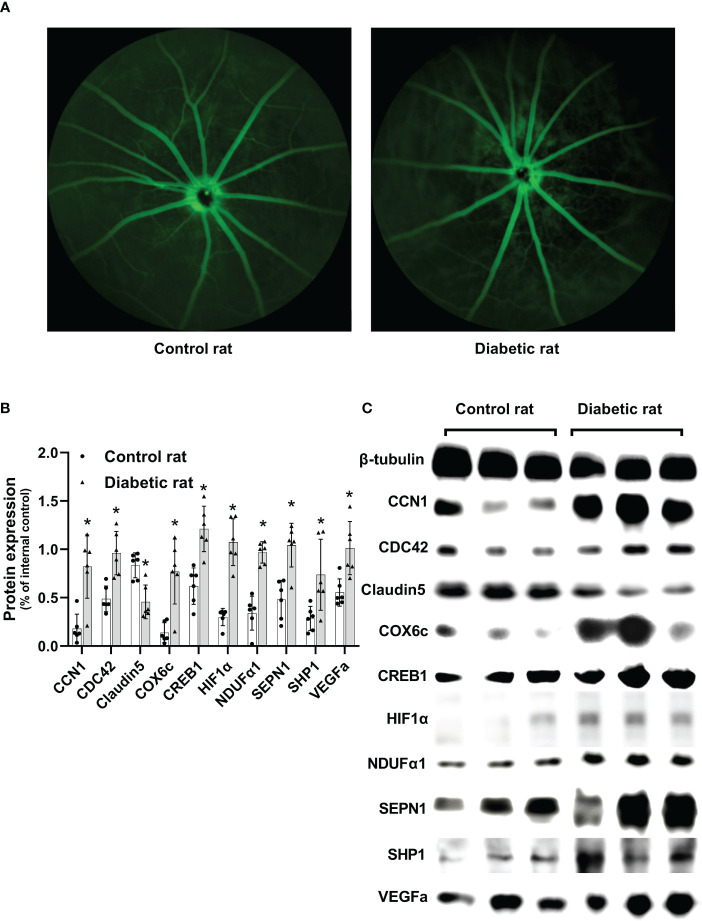
Differences in the retina between diabetic and non-diabetic rats. **(A)** Fundus fluorescein angiography of diabetic and non-diabetic rats. **(B)** Differences in protein expression levels between diabetic and non-diabetic rats. **(C)** Western blotting strip images of diabetic and non-diabetic rats. CCN1, Cellular Communication Network Factor 1; COX6c, cytochrome c oxidase subunit 6c; CREB1, Cyclic AMP-responsive element-binding protein1; HIF1α, Hypoxia-inducible factor 1α; NDUFα1, NADH dehydrogenase 1 alpha subcomplex; SEPN1, Selenoprotein N 1; SHP1, Protein-tyrosine phosphatase 1C; VEGFa, vascular endothelial growth factor a; Differences between control and diabetic rats were compared using the Mann–Whitney test, and the values of protein expression were analyzed using β-tubulin as the internal control; ^*^p< 0.05, compared with controls.

To analyze the changes in the retinal vascular system in diabetic rats, an FFA examination was performed. The optic disc is located at the center of the retina, and the retinal vessels radiate around it. Rats in the control group showed a clear fundus and fluorescence. However, eight weeks after DM induction, rats in the diabetic group exhibited retinal vascular tortuosity, dilation, filling defects, and leakage, which were consistent with DR (**Figure 2A**).

### 3.7 Changes in retinal protein associated with CCN1

Protein expression of CCN1 (U = 35, p = 0.004), as well as CCN1-related proteins (CDC42: U = 36, p = 0.002; COX6c: U = 34, p = 0.009; CREB1: U = 36, p = 0.002; HIF1α: U = 36, p = 0.002; NDUFα1: U = 36, p = 0.002; SEPN1: U = 34, p = 0.009; SHP1: U = 32, p = 0.026) were significantly upregulated in the retina of diabetic rats (all p value< 0.05). Additionally, the protein expression of VEGF was upregulated (U = 35, p = 0.004), while Claudin5 was downregulated (U = 34, p = 0.009) in the retina of diabetic rats (**Figures 2B, C**).

## 4 Discussion

DR is the leading cause of blindness in working-age adults (1). CCN1 is upregulated by various stimuli and exerts various biological effects by interacting with the extracellular matrix and cell surface. CCN1 has been shown to be closely related to DR. Moreover, CCN1 in the vitreous humor and neovascular membrane has received considerable attention; however, research on blood levels of CCN1 is limited. Since blood is a more accessible biological sample, the study of blood CCN1 levels could help us further understand the relationship between CCN1 and DR and its potential clinical application. We report, for the first time, the comparison of the blood CCN1 levels between patients with type 2 diabetes and healthy individuals. We observed that plasma CCN1 levels in patients with DR were significantly higher than those in diabetic patients without DR and healthy subjects. This finding is consistent with previous conclusions drawn from studies on vitreous humor.

Winzap et al. analyzed blood CCN1 levels in patients with acute coronary syndrome and established that diabetes does not affect CCN1 levels (23). This observation is consistent with our results, which revealed a non-significant difference in the CCN1 levels of the DM and control groups. HbA1c is an important indicator of long-term glycemic control. Neither the study conducted by Winzap et al. (23) nor our study showed a significant correlation between HbA1c and CCN1 levels. However, Winzap et al. found that baseline blood glucose levels were correlated with CCN1 levels (23), which was not observed in our study. This difference may be due to the inclusion of patients with acute coronary syndrome in their study; additionally, the blood glucose levels may have partially reflected the stress level in the body (23). However, the blood glucose level in our study reflected the subjects’ glycemic control status.

We found that BMI, duration of diabetes, and urea levels were associated with CCN1 expression, but these correlations were weak. Considering the relation between CCN1 and DR, we believe that the correlation between CCN1 and these parameters may be due to their association with DR. CCN1 is closely associated with vascular injury. Feng et al. reported that blood CCN1 levels in diabetic subjects were positively correlated with severity of peripheral arterial disease (6). CCN1 levels is significantly elevated in patients with ST-segment elevation myocardial infarction compared to that in patients with non-ST-segment elevation myocardial infarction (24), and high levels of CCN1 can potentially predict the occurrence of cardiovascular risk events (23–25). We believe that the elevation of CCN1 levels may reflect the impairment of organ function, especially the function of blood vessels. DR is characterized by a series of fundus lesions caused by leakage and occlusion of retinal microvessels. CCN1 may be involved in the development and progression of DR through its effects on the blood vessels.

As an early response gene, CCN1 is induced by various factors, such as inflammation, hypoxia, and mechanical stimulation (2). Changes in *HIF1A-AS3, CDC42-IT1, SRCAP, AC116348.2, CDC42EP1*, and *AL117336.1* in blood mRNA sequencing analysis may reflect the induction of CCN1. Previous studies have shown that hypoxia significantly induced CCN1 expression in endothelial cells (5, 26) and HIF-1α promoted CCN1 transcription under hypoxia (26, 27). In our study, *HIF1A-AS3* was found to be upregulated in DR. Previous studies have revealed that *HIF1A-AS1* and *HIF1A-AS2* regulate the expression of HIF-1α mRNA (28, 29). We observed an increase in HIF1α in diabetic rats, which reflects the hypoxic state of the retina. In addition, changes in the expression of *CDC42-IT1, SRCAP, CDC42EP1*, and *AL117336.1* may indicate changes in CREB and small GTPase-related pathways, which have been shown to play a key role in the regulation of CCN1 (30–32). The phosphorylation of CREB and its kinases play a vital role in RhoA-mediated regulation (30, 31, 33). The *SRCAP*, which is upregulated in the DR group, is a transcriptional activator of CREB-mediated transcription (34). *CDC42EP1* is a CDC42-binding protein that promotes angiogenesis through cytoskeletal regulation (35). Additionally, *CDC42-IT1* and *AL117336.1* may be involved in the regulation of CREB-related pathways; however, their specific roles remain unclear. CDC42 and CREB1 levels were higher in the retina of diabetic rats. CREB regulates the transcription of CCN1 by directly binding to its promoter (30, 31). *AC116348.2* encodes an integrin-related molecule, and it has been reported that secreted CCN1 exerts its biological effects by binding to integrins (36).

High plasma levels of CCN1 in patients might indicate an impaired blood-retinal barrier. Changes in *SELENON, MIF, AC009927.1, COX6CP1, AC112191.1, NOG*, and *AL157871.3* in blood mRNA sequencing analysis indicated changes in systemic oxidative stress or phosphorylation levels, suggesting the possibility of blood-retinal barrier injury. Diabetic rats showed vascular tortuosity and leakage upon FFA examination. VEGF expression increased in the retinas of diabetic rats, while Claudin5 expression decreased. The retinal vessels of diabetic rats were damaged and the blood-retinal barrier was compromised. CCN1 is mainly expressed in endothelial cells and mediates angiogenic effects (36). Previous studies have suggested that increased CCN1 expression stimulates oxidative stress and disrupts tight junction integrity in endothelial cells (13). In our study, alterations in the expression of *SELENON, COX6CP1, NOG*, and *AL157871.3* suggest elevated levels of oxidative stress. We examined the relevant target molecules and found that the target molecules COX6c, NDUFα1, and SEPN1 levels were elevated in the retinas of diabetic rats. In addition, CCN1 can inhibit pericyte adhesion and cause anoikis by regulating the phosphorylation of focal adhesions (37). *AC009927.1* and *AC112191.1* are associated with the regulation of phosphorylation, particularly tyrosine phosphorylation. We examined the levels of SHP1, which promotes dephosphorylation, in the retina and found that SHP1 was upregulated in diabetic rats, consistent with previous studies (37).

This study has some limitations. First, this was a cross-sectional study, and longitudinal cohorts are needed to demonstrate the predictive effect of CCN1 on DR. Second, we did not differentiate the severity of DR and, therefore, could not clarify the relation between CCN1 levels and the severity of DR. Third, our study included only subjects from Shanghai, China, and the generalizability of the findings remains to be proven. Fourth, we discussed the mechanism of CCN1 based only on sequencing and retinal protein results, which provides information for subsequent mechanistic studies that need to be verified in the future. Fifth, we did not test the level of CCN1 in the vitreous fluid and could not analyze the relationship between CCN1 vitreous levels and CCN1 blood levels.

In conclusion, we measured plasma CCN1 levels in the healthy controls, DM, and DR groups and found that CCN1 expression was significantly elevated in patients with DR, whereas there was no difference between healthy controls and DM groups. Additionally, it was observed that elevated CCN1 levels were risk factors for DR, even after multifactorial correction. CCN1 was negatively correlated with BMI and positively correlated with the duration of diabetes and urea levels. Thus, CCN1 levels may reflect vascular damage caused by chronic hyperglycemia, rather than glycemic control. The results of mRNA sequencing analysis suggested that CCN1 levels in the blood could be upregulated due to hypoxia and may damage the blood-retinal barrier through oxidative stress and phosphorylation after interaction with integrin. Blood CCN1 levels may be a potential biomarker for DR and may be involved in the occurrence and development of DR.

## Data availability statement

The datasets presented in this study can be found in online repositories. The names of the repository/repositories and accession number(s) can be found below: https://www.ncbi.nlm.nih.gov/geo/, GSE221521.

## Ethics statement

The studies involving human participants were reviewed and approved by the Ethics Committee of Shanghai First People’s Hospital. The patients/participants provided their written informed consent to participate in this study. The animal study was reviewed and approved by Shanghai First People’s Hospital Animal Ethics Committee.

## Author contributions

ZX contributes to the acquisition of data, analysis, and interpretation of data, and drafting the manuscript. SC, XQ, SL, YX and LL contributes to the acquisition of data and interpretation of data. HZ contributes to the conception, design, and final approval. All authors read and approved the manuscript.

## References

[1] Zheng Y He M Congdon N. The worldwide epidemic of diabetic retinopathy. Indian J Ophthalmol (2012) 60(5):428–31. doi: 10.4103/0301-4738.100542 PMC3491270 22944754

[2] Kim KH Won JH Cheng N Lau LF. The matricellular protein Ccn1 in tissue injury repair. J Cell Commun Signal (2018) 12(1):273–9. doi: 10.1007/s12079-018-0450-x PMC5842204 29357009

[3] Lee S Elaskandrany M Lau LF Lazzaro D Grant MB Chaqour B. Interplay between Ccn1 and Wnt5a in endothelial cells and pericytes determines the angiogenic outcome in a model of ischemic retinopathy. Sci Rep (2017) 7(1):1405. doi: 10.1038/s41598-017-01585-8 28469167 PMC5431199

[4] Hughes JM Kuiper EJ Klaassen I Canning P Stitt AW Van Bezu J. Advanced glycation end products cause increased ccn family and extracellular matrix gene expression in the diabetic rodent retina. Diabetologia (2007) 50(5):1089–98. doi: 10.1007/s00125-007-0621-4 PMC1914292 17333105

[5] You JJ Yang CH Chen MS Yang CM. Cysteine-rich 61, a member of the ccn family, as a factor involved in the pathogenesis of proliferative diabetic retinopathy. Invest Ophthalmol Vis Sci (2009) 50(7):3447–55. doi: 10.1167/iovs.08-2603 19264885

[6] Feng B Xu G Sun K Duan K Shi B Zhang N. Association of serum Cyr61 levels with peripheral arterial disease in subjects with type 2 diabetes. Cardiovasc Diabetol (2020) 19(1):194. doi: 10.1186/s12933-020-01171-9 33222686 PMC7680586

[7] Ge K Wu JJ Qian L Wu MJ Wang FL Xu B. Bioinformatic analysis of the effect of type ii diabetes on skin wound healing. Genet Mol Res (2015) 14(2):4802–11. doi: 10.4238/2015.May.11.12 25966254

[8] Sawai K Mukoyama M Mori K Kasahara M Koshikawa M Yokoi H. Expression of Ccn1 (Cyr61) in developing, normal, and diseased human kidney. Am J Physiol Renal Physiol (2007) 293(4):F1363–72. doi: 10.1152/ajprenal.00205.2007 17699553

[9] Zhang X Yu W Dong F. Cysteine-rich 61 (Cyr61) is up-regulated in proliferative diabetic retinopathy. Graefes Arch Clin Exp Ophthalmol (2012) 250(5):661–8. doi: 10.1007/s00417-011-1882-7 22160564

[10] Choi J Lin A Shrier E Lau LF Grant MB Chaqour B. Degradome products of the matricellular protein Ccn1 as modulators of pathological angiogenesis in the retina. J Biol Chem (2013) 288(32):23075–89. doi: 10.1074/jbc.M113.475418 PMC3743481 23798676

[11] Zhou F Zhang Y Chen D Su Z Jin L Wang L. Potential role of Cyr61 induced degeneration of human Muller cells in diabetic retinopathy. PloS One (2014) 9(10):e109418. doi: 10.1371/journal.pone.0109418 25329584 PMC4199605

[12] You JJ Yang CM Chen MS Yang CH. Elevation of angiogenic factor cysteine-rich 61 levels in vitreous of patients with proliferative diabetic retinopathy. Retina (2012) 32(1):103–11. doi: 10.1097/IAE.0b013e318219e4ad 21822163

[13] Li H Li T Wang H He X Li Y Wen S. Diabetes promotes retinal vascular endothelial cell injury by inducing Ccn1 expression. Front Cardiovasc Med (2021) 8:689318. doi: 10.3389/fcvm.2021.689318 34458333 PMC8385274

[14] Kuiper EJ Hughes JM Van Geest RJ Vogels IM Goldschmeding R Van Noorden CJ. Effect of vegf-a on expression of profibrotic growth factor and extracellular matrix genes in the retina. Invest Ophthalmol Vis Sci (2007) 48(9):4267–76. doi: 10.1167/iovs.06-0804 17724216

[15] Sun L Huang T Xu W Sun J Lv Y Wang Y. Advanced glycation end products promote vegf expression and thus choroidal neovascularization *Via* Cyr61-Pi3k/Akt signaling pathway. Sci Rep (2017) 7(1):14925. doi: 10.1038/s41598-017-14015-6 29097668 PMC5668426

[16] Di Y Zhang Y Nie Q Chen X. Ccn1/Cyr61-Pi3k/Akt signaling promotes retinal neovascularization in oxygen-induced retinopathy. Int J Mol Med (2015) 36(6):1507–18. doi: 10.3892/ijmm.2015.2371 PMC4678165 26459773

[17] Di Y Zhang Y Yang H Wang A Chen X. The mechanism of Ccn1-enhanced retinal neovascularization in oxygen-induced retinopathy through Pi3k/Akt-vegf signaling pathway. Drug Des Devel Ther (2015) 9:2463–73. doi: 10.2147/DDDT.S79782 PMC4425238 25995618

[18] Wang N Xu X Zou H Zhu J Wang W Ho PC. The status of diabetic retinopathy and diabetic macular edema in patients with type 2 diabetes: a survey from beixinjing district of shanghai city in China. Ophthalmologica (2008) 222(1):32–6. doi: 10.1159/000109276 18097178

[19] Peng J Zou H Wang W Fu J Shen B Bai X. Implementation and first-year screening results of an ocular telehealth system for diabetic retinopathy in China. BMC Health Serv Res (2011) 11:250. doi: 10.1186/1472-6963-11-250 21970365 PMC3200176

[20] Lin Q Jia Y Li T Wang S Xu X Xu Y. Optic disc morphology and peripapillary atrophic changes in diabetic children and adults without diabetic retinopathy or visual impairment. Acta Ophthalmol (2022) 100(1):e157–e66. doi: 10.1111/aos.14885 PMC9292269 33949131

[21] Kuzuya T. Early diagnosis, early treatment and the new diagnostic criteria of diabetes mellitus. Br J Nutr (2000) 84 Suppl 2:S177–81. doi: 10.1079/096582197388644 11242465

[22] Wilkinson CP Ferris FL 3rd Klein RE Lee PP Agardh CD Davis M. Proposed international clinical diabetic retinopathy and diabetic macular edema disease severity scales. Ophthalmology (2003) 110(9):1677–82. doi: 10.1016/s0161-6420(03)00475-5 13129861

[23] Winzap P Davies A Klingenberg R Obeid S Roffi M Mach F. Diabetes and baseline glucose are associated with inflammation, left ventricular function and short- and long-term outcome in acute coronary syndromes: role of the novel biomarker cyr 61. Cardiovasc Diabetol (2019) 18(1):142. doi: 10.1186/s12933-019-0946-6 31672144 PMC6824030

[24] Klingenberg R Aghlmandi S Liebetrau C Räber L Gencer B Nanchen D. Cysteine-rich angiogenic inducer 61 (Cyr61): a novel soluble biomarker of acute myocardial injury improves risk stratification after acute coronary syndromes. Eur Heart J (2017) 38(47):3493–502. doi: 10.1093/eurheartj/ehx640 29155984

[25] Liu C Cao Y He X Zhang C Liu J Zhang L. Association of Cyr61-Cysteine-Rich protein 61 and short-term mortality in patients with acute heart failure and coronary heart disease. biomark Med (2019) 13(18):1589–97. doi: 10.2217/bmm-2019-0111 31660756

[26] You JJ Yang CM Chen MS Yang CH. Regulation of Cyr61/Ccn1 expression by hypoxia through cooperation of c-Jun/Ap-1 and hif-1alpha in retinal vascular endothelial cells. Exp Eye Res (2010) 91(6):825–36. doi: 10.1016/j.exer.2010.10.006 21029732

[27] Kunz M Moeller S Koczan D Lorenz P Wenger RH Glocker MO. Mechanisms of hypoxic gene regulation of angiogenesis factor Cyr61 in melanoma cells. J Biol Chem (2003) 278(46):45651–60. doi: 10.1074/jbc.M301373200 12939282

[28] Li L Wang M Mei Z Cao W Yang Y Wang Y. Lncrnas Hif1a-As2 facilitates the up-regulation of hif-1α by sponging to mir-153-3p, whereby promoting angiogenesis in huvecs in hypoxia. BioMed Pharmacother (2017) 96:165–72. doi: 10.1016/j.biopha.2017.09.113 28985553

[29] Chen D Wu L Liu L Gong Q Zheng J Peng C. Comparison of Hif1a−As1 and Hif1a−As2 in regulating Hif−1α and the osteogenic differentiation of pdlcs under hypoxia. Int J Mol Med (2017) 40(5):1529–36. doi: 10.3892/ijmm.2017.3138 28949371

[30] Hanna M Liu H Amir J Sun Y Morris SW Siddiqui MA. Mechanical regulation of the proangiogenic factor Ccn1/Cyr61 gene requires the combined activities of mrtf-a and creb-binding protein histone acetyltransferase. J Biol Chem (2009) 284(34):23125–36. doi: 10.1074/jbc.M109.019059 PMC2755718 19542562

[31] Han JS Macarak E Rosenbloom J Chung KC Chaqour B. Regulation of Cyr61/Ccn1 gene expression through rhoa gtpase and P38mapk signaling pathways. Eur J Biochem (2003) 270(16):3408–21. doi: 10.1046/j.1432-1033.2003.03723.x 12899698

[32] Walsh CT Radeff-Huang J Matteo R Hsiao A Subramaniam S Stupack D. Thrombin receptor and rhoa mediate cell proliferation through integrins and cysteine-rich protein 61. FASEB J (2008) 22(11):4011–21. doi: 10.1096/fj.08-113266 PMC3980657 18687805

[33] Dobroff AS Wang H Melnikova VO Villares GJ Zigler M Huang L. Silencing camp-response element-binding protein (Creb) identifies Cyr61 as a tumor suppressor gene in melanoma. J Biol Chem (2009) 284(38):26194–206. doi: 10.1074/jbc.M109.019836 PMC2758018 19632997

[34] Johnston H Kneer J Chackalaparampil I Yaciuk P Chrivia J. Identification of a novel Snf2/Swi2 protein family member, srcap, which interacts with creb-binding protein. J Biol Chem (1999) 274(23):16370–6. doi: 10.1074/jbc.274.23.16370 10347196

[35] Liu Z Vong QP Liu C Zheng Y. Borg5 is required for angiogenesis by regulating persistent directional migration of the cardiac microvascular endothelial cells. Mol Biol Cell (2014) 25(6):841–51. doi: 10.1091/mbc.E13-09-0543 PMC3952853 24451259

[36] Leu SJ Lam SC Lau LF. Pro-angiogenic activities of Cyr61 (Ccn1) mediated through integrins Alphavbeta3 and Alpha6beta1 in human umbilical vein endothelial cells. J Biol Chem (2002) 277(48):46248–55. doi: 10.1074/jbc.M209288200 12364323

[37] Liu H Yang R Tinner B Choudhry A Schutze N Chaqour B. Cysteine-rich protein 61 and connective tissue growth factor induce deadhesion and anoikis of retinal pericytes. Endocrinology (2008) 149(4):1666–77. doi: 10.1210/en.2007-1415 18187544

