# Supplementary material for: Changes and related factors of blood CCN1 levels in diabetic patients
Source: Front Endocrinol (Lausanne). 2023 Jun 2;14:1131993. doi: 10.3389/fendo.2023.1131993 (PMC10273100; doi:10.3389/fendo.2023.1131993)
Supplement: Supplementary file 1 [file DataSheet_1.pdf]

Supplementary Material

ZhaoYu Xiang[1,2], ShuLi Chen[1,2], XinRan Qin[1,2], SenLin Lin[2], Yi Xu[2], LiNa Lu[2], HaiDong Zou[1,2*]

[1] National Clinical Research Center for Eye Diseases, Department of Ophthalmology, Shanghai General Hospital, School of Medicine, Shanghai Jiao Tong University, Shanghai, China

[2] Shanghai Engineering Center for Precise Diagnosis and Treatment of Eye Diseases, Shanghai Eye Diseases Prevention & Treatment Center, Shanghai Eye Hospital, Shanghai, China

*** Correspondence:**
HaiDong Zou
zouhaidong@sjtu.edu.cn

1 Supplementary Results

1.1 Plasma CCN1 in males

In males, blood CCN1 levels were significantly different in the three groups ($H = 10.08$, $p = 0.006$). CCN1 levels in the DR group were significantly higher than those in the DM and the control groups (both $p < 0.05$). However, no significant difference in CCN1 was observed between the control group and the DM group ($p > 0.05$).

1.2 Plasma CCN1 in females

In females, blood CCN1 levels were significantly different in the three groups ($H = 13.01$, $p < 0.001$). CCN1 levels in the DR group were significantly higher than those in the DM and the control groups (both $p < 0.01$). However, no significant difference in CCN1 was observed between the control group and the DM group ($p > 0.05$)

2 Supplementary Figures and Tables

2.1 Supplementary Figures

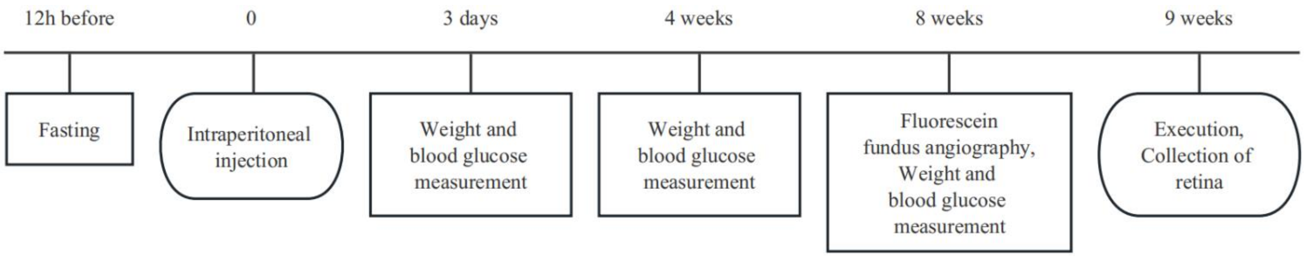

Supplementary Figure 1. Flow chart of animal experiment

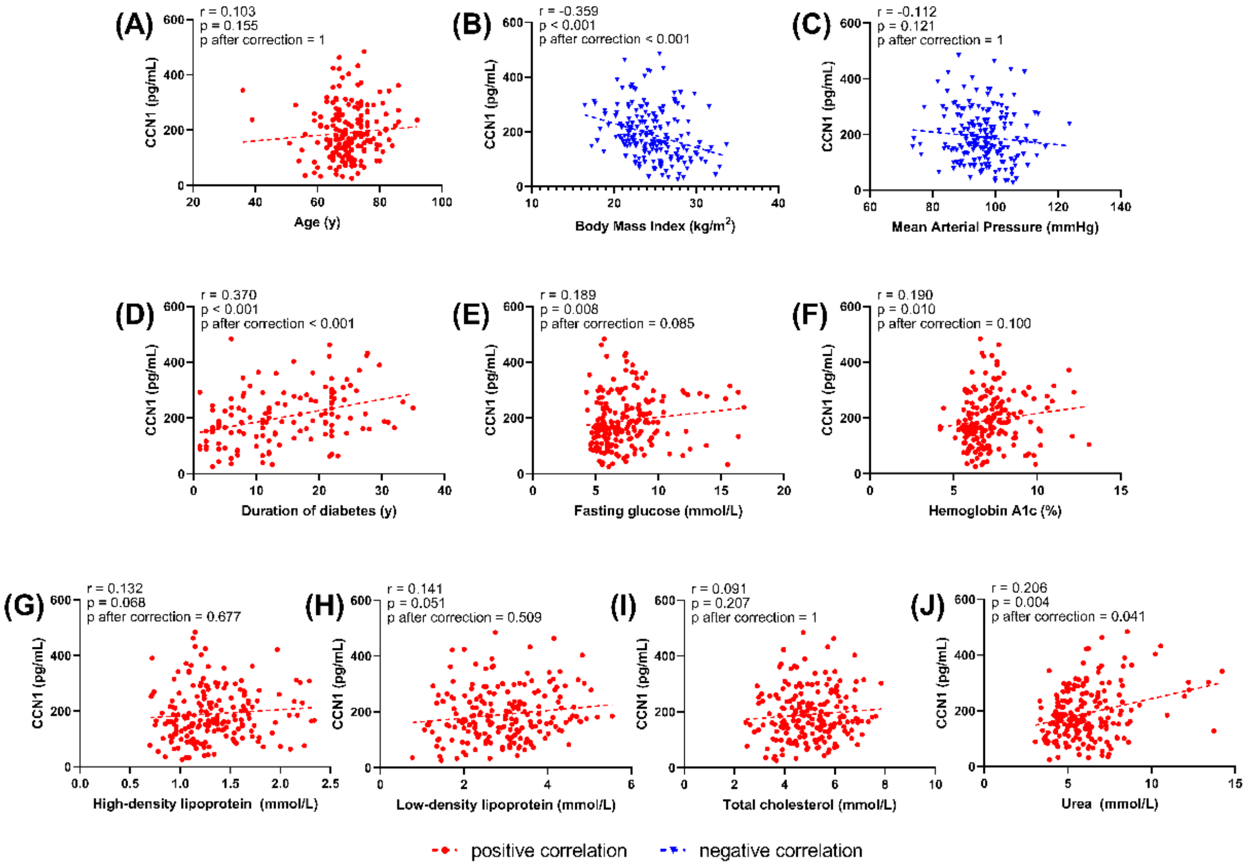

Supplementary Figure 2. Correlation of CCN1 and its influencing factors. **(A)** Correlation between CCN1 and Age. **(B)** Correlation between CCN1 and Body mass index. **(C)** Correlation between CCN1 and Mean arterial pressure. **(D)** Correlation between CCN1 and Duration of diabetes. **(E)** Correlation between CCN1 and Fasting glucose. **(F)** Correlation between CCN1 and Hemoglobin A1c. **(G)** Correlation between CCN1 and High-density lipoprotein. **(H)** Correlation between CCN1 and Low-density lipoprotein. **(I)** Correlation between CCN1 and Total cholesterol. **(J)** Correlation between CCN1 and Urea.

CCN1, Cellular Communication Network Factor 1; Correlation coefficients were calculated by Spearman’s correlation, and multiple comparisons were corrected by Bonferroni's test; red dots indicate positive correlations, and blue triangles indicate negative correlations.

2.2. Supplementary Tables

Supplementary Table 1. Differences in blood mRNA expression associated with CCN1 in DR and control groups

Gene name	Gene ID	Description	Gene type	DR	Control	q-value
HIF1A-AS3	ENSG00000258667	HIF1A antisense RNA 3	lncRNA	1.497	0.733	1.57E-05
SELENON	ENSG00000162430	selenoprotein N	Protein coding	8.964	5.918	3.57E-05
MIF	ENSG00000240972	macrophage migration inhibitory factor	Protein coding	1.059	0.705	3.94E-03
CDC42-IT1	ENSG00000230068	CDC42 intronic transcript 1	lncRNA	1.105	0.552	1.21E-06
SRCAP	ENSG00000080603	Snf2 related CREBBP activator protein	Protein coding	10.995	7.064	1.96E-04
AC009927.1	ENSG00000250031	protein tyrosine phosphatase non-receptor type 11 pseudogene	Processed pseudogene	1.368	0.642	1.33E-04
AC116348.2	ENSG00000261332	antisense to ITGAL	lncRNA	2.025	1.258	4.83E-04
CDC42EP1	ENSG00000128283	CDC42 effector protein 1	Protein coding	2.392	1.402	3.25E-03
COX6CP1	ENSG00000260318	cytochrome c oxidase subunit 6C pseudogene 1	Processed pseudogene	1.244	0.793	3.57E-02
AC112191.1	ENSG00000253772	acid phosphatase 1, soluble pseudogene	Processed pseudogene	1.435	0.772	6.17E-03
AL117336.1	ENSG00000269952	sense intronic to CREM	lncRNA	0.796	1.392	6.07E-04
NOG	ENSG00000183691	noggin	Protein coding	1.362	2.289	3.01E-03
AL157871.3	ENSG00000258581	NADH dehydrogenase 1 β subcomplex pseudogene	Processed pseudogene	2.214	1.102	2.25E-04

CCN1, Cellular Communication Network Factor 1; DR, Diabetic Retinopathy

Supplementary Table 2. Differences in blood mRNA expression associated with CCN1 in DR and DM groups

Gene name	Gene ID	Description	Gene type	DR	DM	q-value
HIF1A-AS3	ENSG00000258667	HIF1A antisense RNA 3	lncRNA	1.497	0.804	3.22E-07
SELENON	ENSG00000162430	selenoprotein N	Protein coding	8.964	5.937	2.19E-06
MIF	ENSG00000240972	macrophage migration inhibitory factor	Protein coding	1.059	0.644	1.57E-05
CDC42-IT1	ENSG00000230068	CDC42 intronic transcript 1	lncRNA	1.105	0.65	2.17E-05
SRCAP	ENSG00000080603	Snf2 related CREBBP activator protein	Protein coding	10.995	7.264	5.25E-05
AC009927.1	ENSG00000250031	protein tyrosine phosphatase non-receptor type 11 pseudogene	Processed pseudogene	1.368	0.767	2.40E-04
AC116348.2	ENSG00000261332	antisense to ITGAL	lncRNA	2.025	1.321	7.36E-04
CDC42EP1	ENSG00000128283	CDC42 effector protein 1	Protein coding	2.392	1.418	1.05E-03
COX6CP1	ENSG00000260318	cytochrome c oxidase subunit 6C pseudogene 1	Processed pseudogene	1.244	0.716	2.14E-03
AC112191.1	ENSG00000253772	acid phosphatase 1, soluble pseudogene	Processed pseudogene	1.435	0.832	4.83E-03
AL117336.1	ENSG00000269952	sense intronic to CREM	lncRNA	0.796	1.302	5.97E-03
NOG	ENSG00000183691	noggin	Protein coding	1.362	2.139	1.09E-02
AL157871.3	ENSG00000258581	NADH dehydrogenase 1 β subcomplex pseudogene	Processed pseudogene	2.214	1.414	3.89E-02

CCN1, Cellular Communication Network Factor 1; DM, Diabetes metillus; DR, Diabetic Retinopathy
